# Commentary: Dimensions of emotional intelligence related to physical and mental health and to health behaviors

**DOI:** 10.3389/fpsyg.2016.00441

**Published:** 2016-03-31

**Authors:** Pablo Fernández-Berrocal, Rosario Cabello

**Affiliations:** ^1^Department of Basic Psychology, Faculty of Psychology, University of MálagaMálaga, Spain; ^2^Department of Evolutionary and Educational Psychology, Faculty of Psychology, University of GranadaGranada, Spain

**Keywords:** emotional intelligence, mental health, physical health, health protective behavior, gender differences

Emotional intelligence (EI) measured through mixed and ability self-report has been related to a broad range of health-related outcomes, including mental and physical health (e.g., Martins et al., 2010; Zeidner et al., 2012). Most of these studies have looked at whether overall self-report EI correlates with health, leaving open the question of whether specific dimensions of self-report EI correlate with specific health dimensions.

The recent article by Fernández-Abascal and Martín-Díaz (2015) begins to address this gap by examining possible relationships between various dimensions of self-report EI and several physical and mental health dimensions and behaviors. Using a cross-sectional sample of Spanish adults (*N* = 855) aged 18–64 years (*M* = 34.27, *SD* = 9.61), the authors applied the two most frequently used instruments (Martins et al., 2010) to evaluate various dimensions of self-report EI. The Trait Meta-Mood Scale (TMMS; Salovey et al., 1995) examines Attention to Feelings, Clarity of Feelings, and Mood Repair; while the Trait Emotional Intelligence Questionnaire (TEIQue; Petrides and Furnham, 2003) examines Well-being, Self-control, Emotionality, and Sociability. The authors examine whether these dimensions are linked to any of several health components, including Physical and Mental Health on the Health Survey SF-36 Questionnaire (SF-36), as well as Preventive Health Behavior and Risk-Taking Behavior on the Health Behavior Checklist (HBC).

Their results suggest that the dimensions of self-report EI in their study are better predictors of Mental Health (48.4%) than of Physical Health (15.6%). Even after controlling for gender and age, Physical and Mental Health correlated positively with Well-Being, Self-Control and Sociability, while Mental Health correlated negatively with Attention to Feelings. Preventive Health Behavior correlated positively with Mood Repair, while Risk-Taking Behavior correlated negatively with Self-control. Effect sizes were smaller for HBC dimensions (ranges from 8.3 to 9.3%) than for SF-36 ones (ranges from 48.4 to 15.6%).

These findings help clarify the relative importance of different dimensions of self-report EI in the prediction of health outcomes. Overall, the authors' results confirm previous research associating the ability to regulate one's emotions (Repair and Self-Control factors in this study) with health outcomes (e.g., Fernández-Berrocal and Extremera, 2008; Freudenthaler et al., 2008; Martins et al., 2010). On the other hand, the results with Physical Health contrast with previous work identifying the ability to understand one's emotions as the self-report EI dimension that most strongly predicts physical health (Mikolajczak et al., 2015). This discrepancy may be due in part to the fact that Fernández-Abascal and Martín-Díaz (2015) measured physical health using self-report instruments, whereas Mikolajczak et al. (2015) measured objective health indicators.

While the results of Fernández-Abascal and Martin-Diaz are interesting in themselves, they are even more interesting because of the questions they raise. The observed association between specific dimensions of self-report EI and health is likely to be the product of multiple biopsychosocial factors. Future studies should examine, for example, how self-report EI interacts with other factors to influence health-related variables such as anxiety, depression, perceived stress, risky behavior and substance abuse. These “other factors” may include socio-demographic variables, such as gender, age, and educational background (Cabello et al., 2014); as well as psychological dimensions, such as implicit theories, social relationships and personality (Cabello and Fernández-Berrocal, 2015a,b). Since self-report measures of EI and performance-based ability measures of EI are complementary, future studies may also wish to examine whether the two types of measures interact to influence health outcomes.

Clearly, detailed understanding of how EI affects health outcomes is just beginning to emerge, and the work of Fernández-Abascal and Martin-Diaz has moved us closer to this goal. If prospective studies can confirm the hypothesis that increasing EI can improve health, it would have significant practical and social implications for training programs aimed at increasing social and emotional competencies in patient groups as well as the general population.

## Author contributions

PF: Substantial contributions to the conception of the work and interpretation of data for the work; Revising the work critically for important intellectual content; Final approval of the version to be published and Agreement to be accountable for all aspects of the work in ensuring that questions related to the accuracy or integrity of any part of the work are appropriately investigated and resolved. RC: Substantial contributions to the conception of the work and interpretation of data for the work; Revising the work critically for important intellectual content; Final approval of the version to be published and Agreement to be accountable for all aspects of the work in ensuring that questions related to the accuracy or integrity of any part of the work are appropriately investigated and resolved.

## Funding

This research was financed by the Spanish Ministry of Economy (PSI2012-37490), and Innovation and Development Agency of Andalusia, Spain (SEJ-07325).

### Conflict of interest statement

The authors declare that the research was conducted in the absence of any commercial or financial relationships that could be construed as a potential conflict of interest.
